# Improvement in the Reproducibility and Accuracy of DNA Microarray Quantification by Optimizing Hybridization Conditions

**DOI:** 10.1186/1471-2105-7-S2-S17

**Published:** 2006-09-26

**Authors:** Tao Han, Cathy D Melvin, Leming Shi, William S Branham, Carrie L Moland, P Scott Pine, Karol L Thompson, James C Fuscoe

**Affiliations:** 1 Center for Functional Genomics, National Center for Toxicological Research, U.S. FDA, Jefferson, AR 72079, USA; 2 Division of Systems Toxicology, National Center for Toxicological Research, U.S. FDA, Jefferson, AR 72079, USA; 3 Center for Drug Evaluation and Research, U.S. FDA, Silver Spring, MD 20993, USA

## Abstract

**Background:**

DNA microarrays, which have been increasingly used to monitor mRNA transcripts at a global level, can provide detailed insight into cellular processes involved in response to drugs and toxins. This is leading to new understandings of signaling networks that operate in the cell, and the molecular basis of diseases. Custom printed oligonucleotide arrays have proven to be an effective way to facilitate the applications of DNA microarray technology. A successful microarray experiment, however, involves many steps: well-designed oligonucleotide probes, printing, RNA extraction and labeling, hybridization, and imaging. Optimization is essential to generate reliable microarray data.

**Results:**

Hybridization and washing steps are crucial for a successful microarray experiment. By following the hybridization and washing conditions recommended by an oligonucleotide provider, it was found that the expression ratios were compressed greater than expected and data analysis revealed a high degree of non-specific binding. A series of experiments was conducted using rat mixed tissue RNA reference material (MTRRM) and other RNA samples to optimize the hybridization and washing conditions. The optimized hybridization and washing conditions greatly reduced the non-specific binding and improved the accuracy of spot intensity measurements.

**Conclusion:**

The results from the optimized hybridization and washing conditions greatly improved the reproducibility and accuracy of expression ratios. These experiments also suggested the importance of probe designs using better bioinformatics approaches and the need for common reference RNA samples for platform performance evaluation in order to fulfill the potential of DNA microarray technology.

## Introduction

DNA microarray has become the major tool to study global gene expression profiles in recent years [2,3]. Data from microarray experiments have been successfully used for establishing new pathways and identifying "signature" genes to differentiate cell types [4,5]. Because of the increased use of microarrays to analyze the gene transcriptional response, it is crucial to ensure the reproducibility, reliability, and accuracy of microarray data.

DNA microarray is a very complex process involving many steps, such as probe design, array fabrication, RNA labeling, hybridization and washing, scanning, and data acquisition. Any missteps in the microarray process may lead to noise in the microarray experiment, which would adversely affect any conclusions drawn from the experiment. Various issues have been raised about the reliability and validity of microarray gene expression data [6-8]. For example, sub-optimally designed probes or incorrect probe annotations can lead to unreliable measurements in microarray experiments [9]. At a more fundamental level, a lack of consistency within and between different microarray platforms when the same RNA samples were tested has also been reported [6-8,10-12]. Such reports cast suspicion on microarray results and conclusions. Recent studies have shown, however, that carefully following established protocols, and using robust experimental designs and appropriate analytical methods can reduce the variability in microarray experiments and can result in much higher reproducibility and consistency [13-16]. In addition, there are many technical issues that must be controlled in the fabrication and use of spotted microarrays that can have a dramatic impact on the quality of microarray data [17]. For example, intra-lab consistency can be improved by (1) the optimization of printing conditions such as relative humidity and buffer composition [18,19], (2) the optimization of purification procedures for RNA amplification and labeling [20,21], and (3) using consistent scanner power and voltage settings [22-24].

The fundamental basis of microarray technology is the specific binding (hybridization) of each probe to a labeled complementary target during the hybridization process [25]. The specificity of each oligonucleotide probe is associated with its melting temperature (Tm) and the salt concentration in the hybridization buffer. Well-designed oligonucleotide sets should have a very narrow Tm range to ensure all the probes have very similar hybridization properties under the chosen hybridization condition.

In this paper, we used tissue and mixed tissue RNA samples to assess the effect of hybridization and washing conditions on the microarray expression ratios. The reproducibility and accuracy (specificity) of microarray data were greatly improved with the optimized hybridization and washing conditions. These experiments also suggest that improvements in probe design will improve the reliability of microarray measurements and the ability to extract meaningful information from microarray data.

## Results

### Detection of non-specific binding under manufacturer-recommended hybridization condition

**Figure 1:**
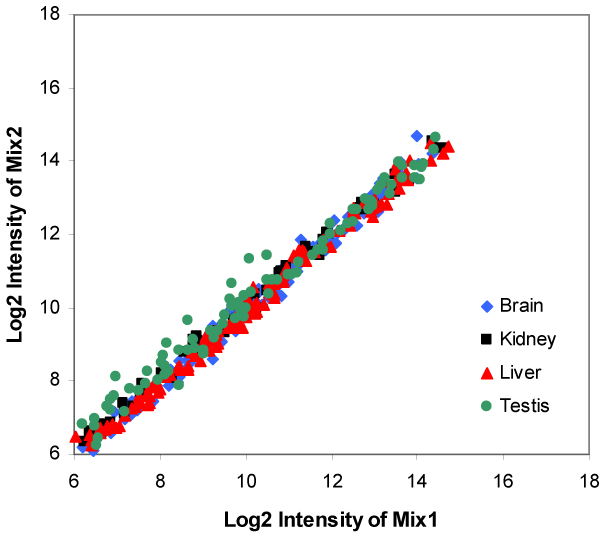
Scatterplot of log2 signal intensities of tissue selective genes from MTRRM Mix1 and Mix2 [1] under non-optimized hybridization and washing conditions.

To evaluate in-house printed Clontech 4 k rat oligonucleotide arrays, rat MTRRM (Mix1 and Mix2; see Materials and Methods) were labeled with cyanine dyes (Cy3 or Cy5) in a flip dye design and hybridized using the GlassHyb™ buffer at 50°C for 16–18 hours. All the slides were washed at room temperature with washing condition 1 (see Materials and Methods). Clontech probes were matched to tissue selective probes on Affymetrix RAE230A arrays based on Unigene ID [26]. Since the signal intensities of these tissue selective genes in the Mix1 and Mix2 samples fall into a wide range, they provide a simple and straightforward tool to use for platform evaluation. The results showed that the expression ratios of the tissue-selective genes under the manufacturer-recommended conditions were greatly compressed compared to the theoretical input ratios (Figure 1; Table 1). It was difficult to distinguish any ratios different from 1 even though the input ratios were 4, 2, 1.5, and 1. The input ratios are easily observed with Affymetrix, Agilent, and Codelink microarray platforms [1]. Thus, this platform, under the manufacturer-recommended conditions, was incapable of reliable measurement of gene expression.

**Figure 2:**
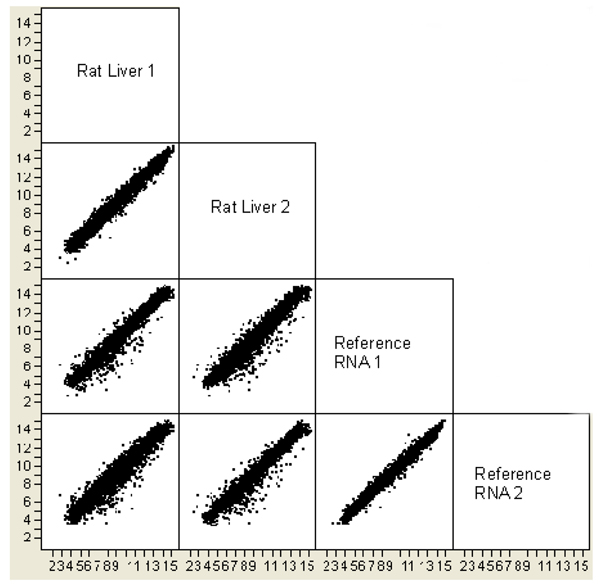
Scatterplots of log2 spot intensities under non-optimized hybridization and washing conditions. Rat liver samples were labeled with Cy5 and Rat Universal Reference RNAs were labeled with Cy3. The labels for the x and y axes are given on the diagonal.

To further investigate the hybridization and washing conditions, rat liver total RNA (Cy 5, NCTR) and Universal Rat Reference RNA (Cy3, Stratagene) were hybridized to the Clontech 4 k rat oligonucleotide arrays under the same hybridization and washing conditions. The log2 intensity correlations (r values) within the rat liver RNAs and within the Universal Rat Reference RNAs were very high, 0.98 and 0.99, respectively, demonstrating high reproducibility (Figure 2). However, correlations between the rat liver RNA and the Universal Rat Reference RNA were also high (0.96–0.97), which indicated that the current hybridization and washing conditions could not effectively detect the differences between the two quite dissimilar RNA samples. The Universal Rat Reference RNA is a blend of RNAs from 14 cell lines, representing 12 tissue types and 2 embryo cell lines, and would be expected to have a mixture of RNAs quantitatively very different from liver RNA. Again, under the manufacturer-recommended hybridization conditions using a second source of dissimilar RNAs, the microarrays were incapable of differential measurement of gene expression levels.

**Figure 3:**
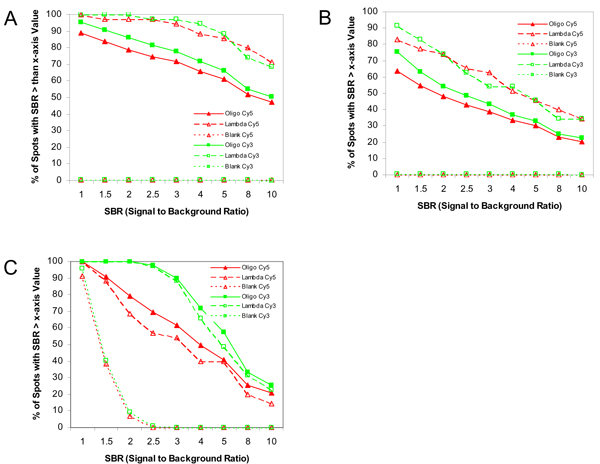
Signal-to-background ratios under different hybridization or washing conditions. A. GlassHyb™ buffer and washing condition 1. B. GlassHyb™ with more stringent washing condition 2. C. 5× SSC, 0.1% SDS and 32% formamide buffer with washing condition 1. Oligo, rat-specific probe; Lambda, bacteriophage lambda-specific probe; Blank, no probe printed. Replicate experiments were conducted and the correlation coefficients were 0.94, 0.97, and 0.97 for conditions shown in panels A, B, and C, respectively.

Further examination of the spot intensities showed that the signals from the bacteriophage lambda control spots were relatively high. Rat RNA would not be expected to hybridize specifically and strongly to lambda probes. The high intensities of lambda spots indicated that there was a high degree of non-specific binding between the labeled cDNA targets and oligonucleotide probes (Figure 3A) and that the hybridization conditions were too relaxed. This would also explain (1) the inability to distinguish the liver RNA sample from the Universal Rat Reference RNA sample, and (2) the highly compressed expression ratio using the rat MTRRM. Alternatively, the probes themselves may have been poorly designed.

### Effect of washing condition stringency on DNA microarray expression ratios

To compare the effect of washing condition alone on the microarray expression ratios, rat liver RNA (Cy5, NCTR) and Universal Rat Reference RNA (Cy3, Stratagene) were hybridized to the 4 k rat oligonucleotide arrays (Clontech) using GlassHyb™ buffer at 50°C for 16–18 hours, but washed differently: one with washing condition 1 and the other with the more stringent washing condition 2 (see Materials and Methods). The results showed that the signal-to-background ratios of lambda spots dropped slightly with the more stringent washing condition (Figures 3A and 3B). However, the lambda spots still gave higher signal-to-background ratios than the rat-specific oligonucleotide probes. Because of the relatively small improvement in signal-to-background ratios as a result of making the washing conditions more stringent, we focused on adjusting the hybridization conditions.

### Effect of hybridization condition on DNA microarray expression ratios

Since the Clontech oligonucleotide probe sequences and the composition of their hybridization buffer GlassHyb™ are proprietary and unknown to us, rat liver RNA (Cy5, NCTR) and Universal Rat Reference RNA (Cy3, Stratagene) were hybridized to the rat 4 k arrays (Clontech) using a hybridization buffer composed of 5× SSC, 0.1% SDS and 32% formamide. The hybridization buffer was defined using the equation Tm = 81.5 + 16.6 (log10 M) + 0.41 (% GC) - 0.61 (% form) - 500/L, where Tm is the melting temperature, M is the molarity of Na^+^, L is the length of base pairs, and % form is the percentage of formamide [25]. It was assumed that the 80-mer oligonucleotide probes from Clontech contained an equal number of A, T, C, and G bases. The hybridization temperature was targeted about 20°C below the Tm. The hybridization temperature was set to 50°C so adjustments were made in the Na^+ ^and formamide concentration to meet these criteria. The slides were washed with washing condition 1. The results (Figure 3C) showed that the stringent hybridization conditions dramatically reduced the signal-to-background ratios of the lambda sequences while maintaining high signal-to-background ratios for the rat sequences. Thus, the hybridization condition appeared to play a greater role than the washing condition in reducing non-specific binding between the labeled targets and the oligonucleotide probes on the microarray.

**Figure 4:**
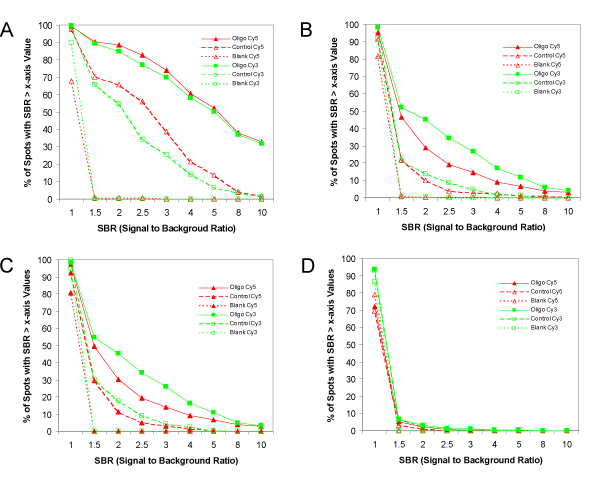
Signal-to-background ratios under different hybridization conditions. Labeled mouse liver RNA (Cy5) and Universal Mouse Reference RNA samples (Cy3) were hybridized with MWG mouse 20 K using GlassHyb™ buffer or 5× SSC, 0.1% SDS buffer with different concentrations of formamide: A. GlassHyb™ buffer. B. 27% formamide. C. 35% formamide. D. 40% formamide. Oligo, mouse-specific probe; Control, Arabidopsis-specific probe; Blank, no probe printed.

Examination of Figure 3C shows that there was still substantial hybridization to the lambda probe sequences (50%–85% had signal-to-background ratios of >3) that would require further optimization. New, better designed probe sets with many more genes had become available and the findings thus far from the Clontech probes were used to optimize the hybridization conditions for a 10,000 (10 k) oligonucleotide rat set and a 20,000 (20 k) oligonucleotide mouse set manufactured by MWG. Mouse 20 k arrays (MWG) were hybridized with mouse liver RNA (Cy5, NCTR) and Universal Mouse Reference RNA (Cy3, Stratagene) using hybridization buffer 5× SSC, 0.1% SDS and formamide at 27%, 35% and 43%, respectively, or GlassHyb™ buffer and washed under washing condition 3 (see Materials and Methods). The hybridizations were performed at 50°C for 16–18 hours. In the MWG probe collections, control oligonucleotides are composed of Arabidopsis sequences and not lambda sequences as in the Clontech oligonucleotide collection. The results showed that the non-specific binding to the Arabidopsis probes decreased with increasing hybridization stringency (Figure 4A,B,C and 4D). Only 5% of the Arabidopsis probes had signal-to-background ratios of >3. The overall spot intensities dropped to very low levels when the hybridization conditions were too stringent (Figure 4D) and there was no discrimination between the Arabidopsis probes and the rat probes. The hybridization buffers containing 27% and 35% formamide allowed the detection of mouse RNAs while reducing the signal from Arabidopsis probes (Figure 4B and 4C).

**Figure 5:**
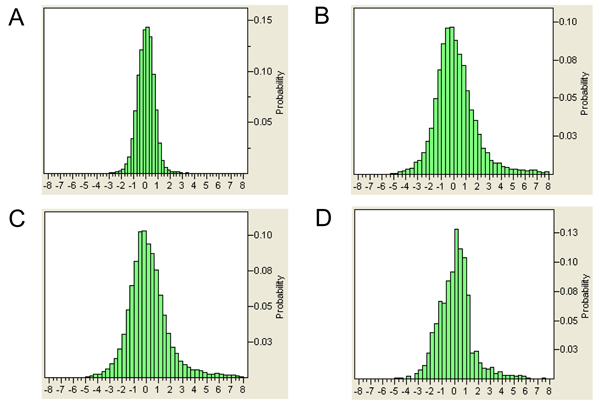
Distribution of log2 expression ratios. Labeled mouse liver RNA (Cy5) and Universal Mouse Reference RNA samples (Cy3) were hybridized with MWG mouse 20 K using GlassHyb™ buffer or 5× SSC, 0.1% SDS buffer with different concentrations of formamide: A. GlassHyb™ buffer. B. 27% formamide. C. 35% formamide. D. 40% formamide. The log2 expression ratio (liver/reference) is plotted on the x-axis and the probability is on the y-axis.

The dynamic ranges of expression ratios (fluorescent intensity from liver RNA/fluorescent intensity from Universal Mouse Reference RNA) followed a similar pattern. At low hybridization stringency, the dynamic range of log2 expression ratios fell within -3 to 3 (Figure 5A) with 90% of ratios less than 2-fold. At higher hybridization stringency, the dynamic range increased to -5 to 8 (Figure 5B and 5C). The dynamic range started to decrease when the hybridization stringency was too high (Figure 5D). The results showed that the hybridization buffer (5× SSC, 0.1% SDS and 27% formamide) offered a good balance between specificity and spot intensity.

**Figure 6:**
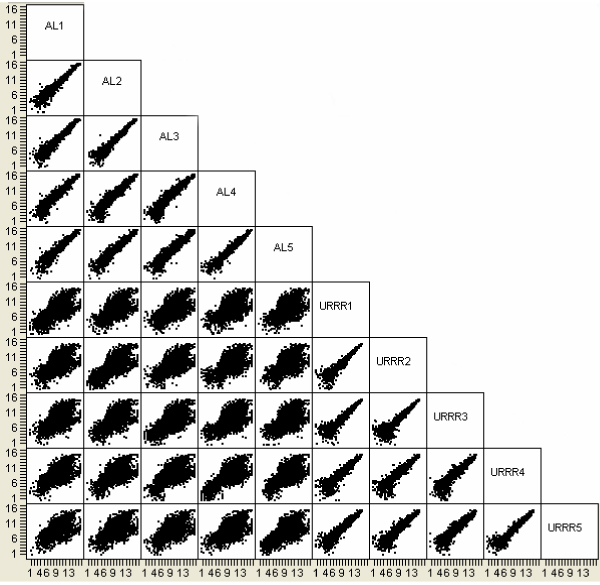
Scatterplots of log2 spot intensities under optimized hybridization and washing conditions. Ambion rat liver RNA samples (AL) were labeled with Cy3 and Stratagene Universal Rat Reference RNAs (URRR) were labeled with Cy5. The labels for the x and y axes are given on the diagonal.

To further evaluate the reproducibility of microarray experiments with this hybridization buffer, rat liver total RNA (Cy5, Ambion), rat brain total RNA (Cy5, Ambion) and Universal Rat Reference RNA (Cy3, Stratagene) were hybridized with MWG rat 10 k arrays using a reference design, and washed with washing condition 3. In this experiment, 5 replicates of rat liver RNA + Universal Rat Reference RNA and 5 replicates of rat brain RNA + Universal Rat Reference RNA were hybridized to the rat 10 k arrays. Figure 6 shows the pair-wise log2 intensity correlations between the 5 liver and 5 Universal Rat Reference RNA samples. There is an obvious good correlation within each sample type (liver or brain) of greater than 0.97 and much lower correlation, as expected, between the 2 dissimilar sample types (r = 0.6). The average log2 ratio (brain or liver/reference) correlation (r) within the brain samples was 0.98 and within the liver samples was 0.97. The log2 ratio correlation between the brain and liver samples was 0.25. The results showed there are very high log2 intensity and ratio correlations within each sample group, indicating excellent reproducibility, and a very good separation between the rat liver total RNA and the universal rat reference RNA, which could not be separated under previous hybridization and washing condition. In addition, 60–70% of the spots had a signal-to-background ratio of greater than 3. The signal-to-background ratio correlation coefficients (r) averaged 0.96 and 0.95 among the 5 liver sample replicates and among the 5 Universal Rat Reference RNA replicates, respectively. Thus, these hybridization conditions appear to allow the quantitative determination of gene expression.

### Discrepancies between expression ratios from microarray and theoretical input ratios, and discrepancies between microarray platforms

**Figure 7:**
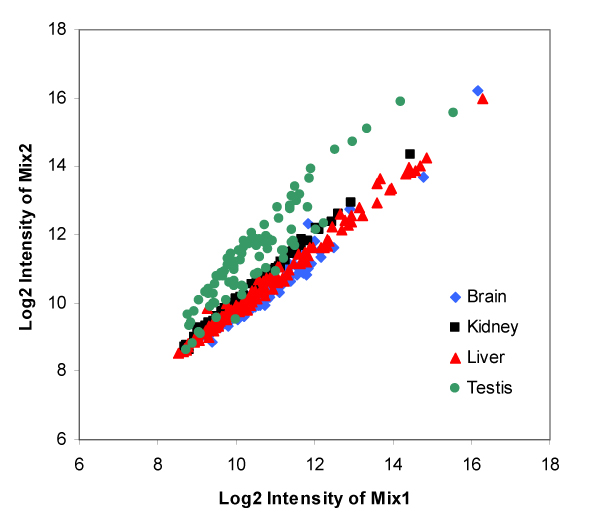
Scatterplot of log2 signal intensities of tissue selective genes from MTRRM Mix1 and Mix2 [1] under optimized hybridization and washing conditions.

**Figure 8:**
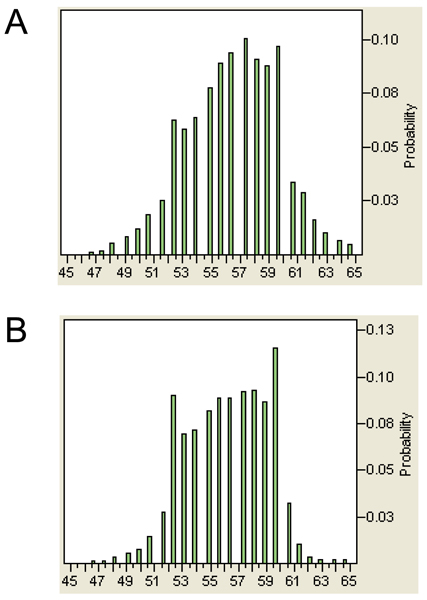
Distribution of Tm's for MWG rat 10 k oligonucleotide (A) and mouse 20 k oligonucleotide (B) probes. The Tm is plotted on the x-axis and the probability on the y-axis.

**Figure 9:**
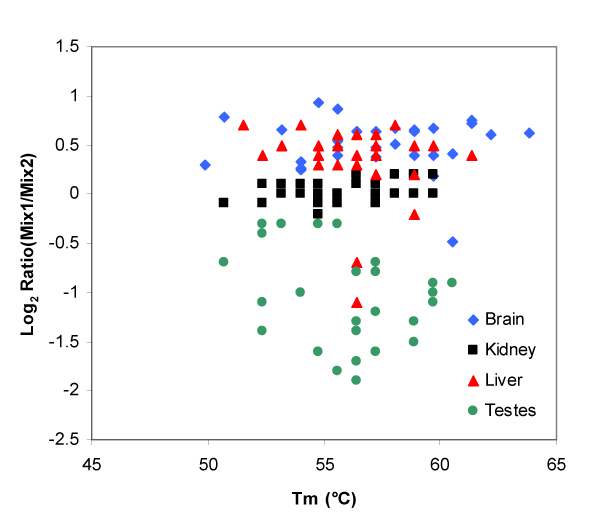
Log2 expression ratios of tissue selective genes vs Tm's of tissue-selective genes in MWG 10 k oligoset. The expected log2 ratios for the brain, kidney, liver, and testes are 1, 0, 0.585, and -2, respectively.

Labeled MTRRM Mix1 and Mix2 were hybridized with rat 10 k arrays under hybridization and washing condition 3 (27% formamide) using a flip dye design. The results showed that the expression ratios of tissue selective genes were greatly improved and much closer to the theoretical input ratios under the optimized hybridization and washing conditions than the one suggested by the oligonucleotide probe provider (compare Figure 1 and Figure 7). However, there were still some discrepancies between expression ratios measured by microarrays compared to the theoretical input ratios. Thompson et al. [1] using the MTRRM samples Mix1 and Mix2, showed close agreement with the input expression ratios on the Affymetrix GeneChip (as well as CodeLink and Agilent microarrays) for probes known to be tissue selective on all 3 platforms. For example, 3 brain-specific genes with input ratio of 2.0 had ratios of 2.02, 2.10, and 2.09 by qRT-PCR. Three testis-specific genes with input ratio of 0.25 had ratios of 0.27, 0.26, and 0.27 by quantitative reverse transcriptase PCR (qRT-PCR). Tissue selectivity was determined empirically based on the relative probe signal level between hybridizations for the individual tissue RNAs in the MTRRM. In the present study, probes in the MWG rat oligonucleotide collections were matched by Unigene ID to a set of probes that were tissue selective on 3 commercial array formats and mapped to a common exemplar sequence. Table 2 shows a comparison of the relative expression levels of 5 transcripts in the MTRRM measured by qRT-PCR and by microarray analysis under the newly optimized conditions. There was reasonably good agreement, although there was compression of the ratios determined by microarray. The MWG (and Clontech) probes were not verified as tissue selective and this may be the cause of the discordant probe behavior. Another possible reason for this discrepancy is that there is a broad range of Tm's for the MWG oligonucleotide probes. The necessary selection of a single hybridization condition will result in non-optimal hybridization conditions for oligonucleotides with Tm's at the fringes of the Tm range. The hypothesis would be that the probes that give the biggest discrepancy between the measured expression ratio and expected ratio (based on the construction of the MTRRM) would be the probes with Tm's far from the median. Figure 8 shows the distributions of the calculated Tm's of the oligonucleotides in the MWG rat 10 k and the MWG mouse 20 k collections. The distribution plots showed that the Tm's of MWG rat 10 k and 20 k oligonucleotides fall into a 10–15°C range, with about 80% within 5°C of the median Tm. Thus, there is a significant number of oligonucleotide probes that would be expected to have hybridization properties different from the average, and whose affinity for targets may be affected. However, there was no obvious correlation between the Tm and the expected log2 expression ratio (Figure 9). The calculated Tm of the probe sequences, therefore, cannot explain the discrepant results.

**Figure 10:**
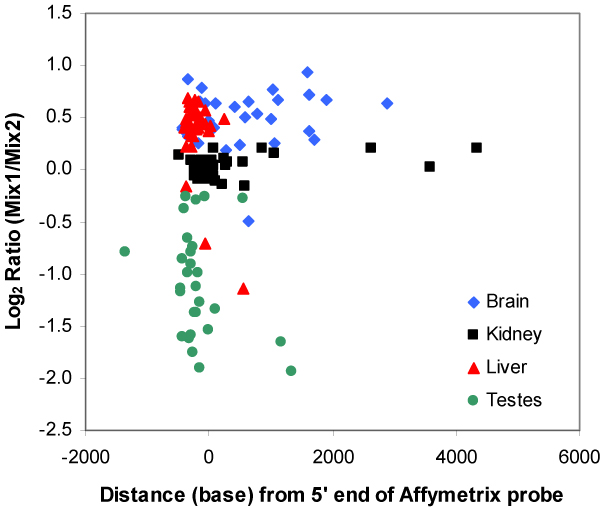
Log2 expression ratios of tissue selective genes vs distances between MWG and Affymetrix probes. The expected log2 ratios for the brain, kidney, liver, and testes are 1, 0, 0.585, and -2, respectively.

Another possible explanation is the Affymetrix and MWG probe sequences are targeted to different parts of the Unigene cluster and that, for example, altered transcript processing may be the cause of the discrepancy. In other words, if the probes come from very different parts of the transcript, they may be measuring different things. Therefore, the MWG and Affymetrix probes were mapped to the transcript and the distance was determined between the probes from different platforms. Figure 10 shows the relationship between the log2 expression ratio and the distance between the Affymetrix and MWG probes for the tissue selective genes identified in the MWG oligonucleotide set. There was no obvious correlation between the expression ratio discrepancies and the probe distance. Thus, there are inconsistencies between microarray platforms (e.g., Affymetrix and MWG) that cannot be explained by Tm or distances between probe sequences.

## Discussion

To assess the performance of our in-house printed oligonucleotide microarrays, mixed tissue RNA reference materials (MTRRM) were labeled and hybridized with rat 4 k arrays under the oligonucleotide manufacturer's recommended condition. The compressed expression ratios for MTRRMs showed there was severe non-specific binding under the recommended hybridization condition. A series of experiments was designed to optimize the hybridization and washing conditions for the in-house printed arrays. With these optimized hybridization and washing conditions, non-specific binding was greatly reduced, and microarray data reproducibility and accuracy were highly improved. Without reference RNA samples, such as the MTRRM, it is very difficult to evaluate accuracy of microarray data. We have shown that very high reproducibility can be obtained under non-optimal hybridization conditions but that the accuracy was very low. The low stringency of the hybridization conditions would cause a failure to identify true changes in gene expression because of the highly compressed nature of the signals due to cross-hybridization. Only through use of calibrated RNA references can the accuracy be judged. The strategy employed by Thompson et al. [1] to generate calibrated rat RNA reference materials can be readily adapted to the production of reference RNA for any desired species, in particular human and mouse. Although discrepancies remained between the ratios measured in this study and the theoretical ratios for the tissue RNA components in the MTRRM, it should be noted that the tissue selectivity of probes recommended for use with the MTRRM was empirically determined for 3 commercial platforms (Agilent, Affymetrix, and CodeLink [1]), but not for the oligonucleotide set used here.

A DNA microarray experiment is a complex, multi-step process. The probe design, hybridization condition, and scanner settings are major factors determining the accuracy and reproducibility of the final specific signal quantification. Signals measured from individual probes on a microarray are a summation of the non-specific hybridization (mismatched cross-hybridization; imperfect matches between probes and targets) and specific hybridization (perfect matches between probes and targets). If the non-specific hybridization signal is relatively large and constant between samples, the ratio measurement between two samples will be compressed as shown in Figure 1. Thus, it is crucial for accurate gene expression measurements to reduce non-specific hybridization as we have described. To avoid non-specific binding (cross-hybridization), probe design has been one of the major focuses for the past few years [27-35]. Commercial microarray manufacturers and oligonucleotide providers have been updating and redesigning their oligonucleotide sets to improve the reliability and quality of the signals. The lack of reference RNA materials, however, has limited the examination of the accuracy of the probes under the conditions recommended by manufacturers.

Aside from probe design, the hybridization stringency (salt concentration, temperature, and pH) plays a critical role to ensure the specific binding of targets to their complementary probes [25]. At a certain hybridization condition, these bindings are not only affected by the Tm's of each probe, but also by the concentration and the secondary structure of the target. This might explain the ratio discrepancies of some of the probes with similar Tm's in our results (Figure 9). The increasing number of probes printed on whole genome microarrays also increases the chance of cross-hybridization (non-specific binding). A recently published study that used a systematic multivariate approach to correct cross-hybridization signals in expression experiments [36] points out the limitations of current understanding of hybridization on solid supports and the consequent difficulty in designing optimal probes.

Recently the discrepancies between various microarray platforms have received great attention in the microarray community. Many studies have been done to address the comparability of different microarray platforms and contradictory conclusions have been reached, with some studies reporting good concordance [13-16] and others a lack of agreement [6-8,10-12]. As in the present study, well-characterized reference RNAs may be valuable in resolving this debate. The FDA-led MicroArray Quality Control (MAQC; ) project is developing such materials and using them to compare the reproducibility of the same microarray platform across sites as well as the comparability between different platforms. The outcome promises to provide a better picture in terms of performances and comparability among different microarray platforms. Meanwhile, development of sets of well-calibrated reference RNA samples, similar to the MTRRMs with expanded gene coverage, would also be useful for microarray performance evaluation. All of these efforts would greatly help to standardize the microarray process and maximize the potential of microarray technology.

## Conclusion

The results from this study demonstrated the importance of hybridization optimization to generate highly reproducible and accurate microarray data. The use of MTRRMs and other dissimilar RNA types were shown to be effective tools for the optimization process.

## Materials and methods

### Microarray slides and sources of RNA samples

Rat 4 k (4000 oligonucleotide probes; Clontech, Palo Alto, CA), rat 10 k (10,000 rat oligonucleotide probes; MWG-Biotech, High Point, NC), and mouse 20 k (20,000 mouse oligonucleotide probes; MWG-Biotech) oligonucleotide sets were dissolved in MWG Spotting Buffer A (MWG-Biotech, High Point, NC) and printed on poly-L-lysine-coated slides (Erie Scientific, Portsmouth, NH) using an OmniGrid™ Microarrayer (GeneMachines, San Carlos, CA) at the National Center for Toxicological Research (NCTR). Printed slides were baked at 80°C for 1 hour and UV cross-linked with 300 mJoules (UV Stratalinker 2400, Stratagene, La Jolla, CA). Following this, the slides were treated with a blocking solution of 3× SSC, 0.1% SDS and 1% BSA using gentle agitation for 5 minutes at 50°C and washed with MilliQ water four times for 5 minutes each at room temperature. The slides were then placed in MilliQ water heated to boiling for 2 minutes, followed by a 1 minute wash in ethanol at room temperature. The slides were spun dry in a microarray high speed centrifuge (TeleChem International, Sunnyvale, CA).

RNA samples used in these experiments were from several different sources; rat liver RNA was extracted at NCTR. Rat mixed tissue RNA reference materials (MTRRM [1]) were provided by the U.S. FDA, Center for Drug Evaluation and Research. Mix1 and Mix2 contain different proportions of four tissue total RNAs. Mix1 contains total RNA from brain (40%), liver (30%), kidney (20%) and testes (10%); Mix2 contains total RNA from brain (20%), liver (20%), kidney (20%) and testes (40%). The theoretical ratios of the tissue selective genes (genes were predominately expressed in only one of the tissues in the mixture) for kidney, liver, brain, and testis between the two mixtures (mix1/mix2) were 1, 1.5, 2, and 0.25, respectively. Universal rat reference RNA (Stratagene, La Jolla, CA), rat brain total RNA and rat liver total RNA (Ambion, Austin, TX) were also used for performance evaluation.

### RNA Isolation and cDNA Labeling

Total RNA was extracted from adult rat and mouse liver tissue using Qiagen RNeasy kits (Qiagen, Valencia, CA) with on-column DNase digestion. The concentrations and A260/A280 ratios of the total RNA samples were measured by NanoDrop ND-1000 spectrophotometry (NanoDrop Technologies, Wilmington, DE). The quality of the total RNA was further evaluated using the Agilent 2100 Bioanalyzer (Agilent Technologies, Palo Alto, CA). RNA samples with RINs (RNA Integrity Number) above 8.0 were used for microarray analysis. All RNA samples were aliquoted at 10 μg per tube and frozen at -80°C until used for microarray analysis.

Target cDNAs were prepared by aminoallyl labeling followed by coupling of fluorescent dyes. Briefly, 10 μg of total RNA and 6 μg of random hexamer primers (Invitrogen, Carlsbad, CA) were mixed together to a final volume of 16.5 μl, incubated at 70°C for 10 minutes and snap frozen on dry-ice and ethanol for 1 minute. The RNA was reverse transcribed in a 30 μl reaction containing 0.5 mM dATP, dCTP, dGTP, 0.3 mM dTTP (Invitrogen), 0.2 mM aminoallyl-dUTP (aa-dUTP, Sigma, St. Louis, MO), 40 U RNase Inhibtor (Invitrogen), 400 U superscript II (Invitrogen), 10 mM DTT and 1X first strand buffer (50 mM Tris-HCl, 75 mM KCl, and 5 mM MgCl_2_, pH 8.3). This mixture was incubated at 42°C for 2 hours to generate aminoallyl-labeled cDNA. The RNA templates were hydrolyzed at 65°C for 15 minutes using 10 μl of 1 N NaOH and 10 μl of 0.5 mM EDTA. The reaction was neutralized by adding 10 μl of 1 N HCl. Unincorporated aa-dUTP was removed by QIAquick PCR purification kit (Qiagen) and the aminoallyl-cDNAs were then dried in a SpeedVac System SPD 1010 (Themo Savant, Holbrook, NY) at 45°C. Aminoallyl-cDNA pellets were resuspended in 4.5 μl of 0.1 M sodium carbonate buffer (pH 9.0) and coupled with 4.5 μl of Cy3 or Cy5 monoreactive dyes (approximately 24 nmoles of dyes) (Amersham Pharmacia, Piscataway, NJ) for 1 hour at room temperature in the dark. Monoreactive Cy3 and Cy5 dyes supplied in each vial were resuspended in 73 μl of DMSO and aliquoted into 16 tubes of 4.5 μl each and one aliquot was used for each labeling reaction. Uncoupled dyes were removed by QIAquick PCR purification kit (Qiagen). cDNA yields and dye incorporation efficiencies were determined using the NanoDrop ND-1000 spectrophotometer.

### Hybridization and washing conditions

Cy3 and Cy5 labeled cDNAs were mixed together and concentrated to less than 5 μl using a SpeedVac SPD 1010 at room temperature. The samples were then mixed with 60 μl of pre-warmed hybridization buffer, either GlassHyb™ (Clontech) or 5× SSC (Ambion), 0.1% SDS (Sigma) with various formamide (Invitrogen) concentrations at 50°C. The labeled cDNAs were denatured at 95°C for 3 minutes and then placed in a water bath at 50°C until hybridization. The hybridizations were performed in ArrayIt hybridization cassettes (TeleChem International) in a water bath at 50°C for 16–18 hours.

Slides were washed under various stringency conditions. Washing condition 1: first wash: 2 × SSC containing 1% Tween-20 (Sigma, St. Louis, MO) at room temperature for 10 minutes on a shaker with gentle agitation; second wash: 1 × SSC containing 0.1% Tween-20 at room temperature for 5–10 minutes on a shaker with gentle agitation; third wash: 0.1 × SSC at room temperature for 5–10 minutes on a shaker with gentle agitation.

Washing condition 2: first wash: 0.3 × SSC containing 1% Tween-20 at 50°C for 10 minutes on a shaker with gentle agitation; second wash: 1 × SSC containing 0.1% Tween-20 at 50°C for 5–10 minutes on a shaker with gentle agitation; third wash: 0.1 × SSC for 5–10 minutes on a shaker with gentle agitation.

Washing condition 3: first wash: 2 × SSC containing 1% SDS at 30°C for 5 minutes on a shaker with gentle agitation; second wash: 1 × SSC at 30°C for 5 minutes on a shaker with gentle agitation; third wash: 0.5 × SSC at 30°C for 5 minutes on a shaker with gentle agitation. The hybridized slides were dried immediately by centrifugation after the final wash step.

### Scanning, Feature Extraction and Data Analysis

The hybridized slides were scanned with a GenePix 4000B scanner (Axon Instruments, Union City, CA) at 10 μm resolution using appropriate photomultiplier tube gains to obtain the highest intensity with <0.1% saturated pixels. The resulting images were analyzed by measuring the fluorescence of all features on the slides using the GenePix Pro 6.0 image analysis software (Axon Instruments). The median fluorescence intensity of all the pixels within one feature was taken as the intensity value for that feature. All the raw data were imported into ArrayTrack [37] and were normalized using Total Intensity Normalization or LOWESS Normalization with background subtraction. Student's T-test was used to compute p values.

## Authors' contributions

TH and JCF participated in the design of experiments and writing the manuscript. WSB and CDM printed all the arrays used in this study. CLM prepared all samples. TH and CDM performed all the microarray experiments for generating the original data. TH, JCF and LS analyzed the data. KLT and PSP provided the sequence mapping information. All authors approved the final manuscript.

## Figures and Tables

**Table 1:** Average log2 ratios (Mix1/Mix2) of tissue selective genes of the mixed tissue RNA reference materials [1].

	Brain (116)	Kidney (66)	Liver (130)	Testis (90)
Old condition	0.12 ± 0.21	0.06 ± 0.22	0.16 ± 0.21	-0.06 ± 0.40
New condition	0.43 ± 0.24	0.01 ± 0.10	0.30 ± 0.21	-0.87 ± 0.58
Affymetrix	1.00 ± 0.28	0.24 ± 0.22	0.65 ± 0.25	-1.31 ± 0.23
Input Ratio	1.00	0.00	0.585	-2.00

Parentheses indicate the total number of tissue selective genes. Affymetrix data is from [1].

**Table 2:** Comparison of transcript expression in MTRRMs (Mix1/Mix2) by qRT-PCR and microarray.

GeneBank number	Gene name	Tissue	Input ratio	qRT-PCR fold-change	Microarray fold-change
NM_012526	*Chgb*	Brain	2	2.02	1.57
NM_020976	*Tmem27*	Kidney	1	0.97	0.94
NM_013042	*Tff3*	Kidney	1	1.13	0.99
NM_057146	*C9*	Liver	1.5	1.47	1.33
NM_080584	*Phkg2*	Testes	0.25	0.27	0.39

The qRT-PCR data is from [1].

## References

[B1] Thompson KL, Rosenzweig BA, Pine PS, Retief J, Turpaz Y, Afshari CA, Hamadeh HK, Damore MA, Boedigheimer M, Blomme E (2005). Use of a mixed tissue RNA design for performance assessments on multiple microarray formats. Nucleic Acids Res.

[B2] Schena M, Shalon D, Davis RW, Brown PO (1995). Quantitative monitoring of gene expression patterns with a complementary DNA microarray. Science.

[B3] Brown PO, Botstein D (1999). Exploring the new world of the genome with DNA microarrays. Nat Genet.

[B4] Huang S (1999). Gene expression profiling, genetic networks, and cellular states: an integrating concept for tumorigenesis and drug discovery. J Mol Med.

[B5] Sgroi DC, Teng S, Robinson G, LeVangie R, Hudson JR, Elkahloun AG (1999). In vivo gene expression profile analysis of human breast cancer progression. Cancer Res.

[B6] Kothapalli R, Yoder SJ, Mane S, Loughran TP (2002). Microarray results: how accurate are they?. BMC Bioinformatics.

[B7] Tan PK, Downey TJ, Spitznagel EL, Xu P, Fu D, Dimitrov DS, Lempicki RA, Raaka BM, Cam MC (2003). Evaluation of gene expression measurements from commercial microarray platforms. Nucleic Acids Res.

[B8] Marshall E (2004). Getting the noise out of gene arrays. Science.

[B9] Draghici S, Khatri P, Eklund AC, Szallasi Z (2006). Reliability and reproducibility issues in DNA microarray measurements. Trends Genet.

[B10] Jarvinen AK, Hautaniemi S, Edgren H, Auvinen P, Saarela J, Kallioniemi OP, Monni O (2004). Are data from different gene expression microarray platforms comparable?. Genomics.

[B11] Rogojina AT, Orr WE, Song BK, Geisert EE (2003). Comparing the use of Affymetrix to spotted oligonucleotide microarrays using two retinal pigment epithelium cell lines. Mol Vis.

[B12] Mah N, Thelin A, Lu T, Nikolaus S, Kuhbacher T, Gurbuz Y, Eickhoff H, Kloppel G, Lehrach H, Mellgard B (2004). A comparison of oligonucleotide and cDNA-based microarray systems. Physiol Genomics.

[B13] Yauk CL, Berndt ML, Williams A, Douglas GR (2004). Comprehensive comparison of six microarray technologies. Nucleic Acids Res.

[B14] Bammler T, Beyer RP, Bhattacharya S, Boorman GA, Boyles A, Bradford BU, Bumgarner RE, Bushel PR, Chaturvedi K, Choi D (2005). Standardizing global gene expression analysis between laboratories and across platforms. Nat Methods.

[B15] Larkin JE, Frank BC, Gavras H, Sultana R, Quackenbush J (2005). Independence and reproducibility across microarray platforms. Nat Methods.

[B16] Irizarry RA, Warren D, Spencer F, Kim IF, Biswal S, Frank BC, Gabrielson E, Garcia JG, Geoghegan J, Germino G (2005). Multiple-laboratory comparison of microarray platforms. Nat Methods.

[B17] Fuscoe JC, Branham WS, Melvin CD, Desai VG, Moland CL, Han T, Shi L, Tong W, Scully AT, Delongchamp RR (2006). Technical Issues Involved in Obtaining Reliable Data from Microarray Experiments. Regulatory Research Perspectives.

[B18] McQuain MK, Seale K, Peek J, Levy S, Haselton FR (2003). Effects of relative humidity and buffer additives on the contact printing of microarrays by quill pins. Anal Biochem.

[B19] Diehl F, Grahlmann S, Beier M, Hoheisel JD (2001). Manufacturing DNA microarrays of high spot homogeneity and reduced background signal. Nucleic Acids Res.

[B20] Lage JM, Hamann S, Gribanov O, Leamon JH, Pejovic T, Lizardi PM (2002). Microgel assessment of nucleic acid integrity and labeling quality in microarray experiments. Biotechniques.

[B21] Naderi A, Ahmed AA, Barbosa-Morais NL, Aparicio S, Brenton JD, Caldas C (2004). Expression microarray reproducibility is improved by optimising purification steps in RNA amplification and labelling. BMC Genomics.

[B22] Wang X, Ghosh S, Guo SW (2001). Quantitative quality control in microarray image processing and data acquisition. Nucleic Acids Res.

[B23] Lyng H, Badiee A, Svendsrud DH, Hovig E, Myklebost O, Stokke T (2004). Profound influence of microarray scanner characteristics on gene expression ratios: analysis and procedure for correction. BMC Genomics.

[B24] Shi L, Tong W, Su Z, Han T, Han J, Puri RK, Fang H, Frueh FW, Goodsaid FM, Guo L (2005). Microarray scanner calibration curves: characteristics and implications. BMC Bioinformatics.

[B25] Meinkoth J, Wahl G (1984). Hybridization of nucleic acids immobilized on solid supports. Anal Biochem.

[B26] Wheeler DL, Church DM, Federhen S, Lash AE, Madden TL, Pontius JU, Schuler GD, Schriml LM, Sequeira E, Tatusova TA (2003). Database resources of the National Center for Biotechnology. Nucleic Acids Res.

[B27] Chen YA, McKillen DJ, Wu S, Jenny MJ, Chapman R, Gross PS, Warr GW, Almeida JS (2004). Optimal cDNA microarray design using expressed sequence tags for organisms with limited genomic information. BMC Bioinformatics.

[B28] Chen DT, Chen JJ, Soong SJ (2005). Probe rank approaches for gene selection in oligonucleotide arrays with a small number of replicates. Bioinformatics.

[B29] Emrich SJ, Lowe M, Delcher AL (2003). PROBEmer: A web-based software tool for selecting optimal DNA oligos. Nucleic Acids Res.

[B30] Nordberg EK (2005). YODA: selecting signature oligonucleotides. Bioinformatics.

[B31] Nielsen HB, Knudsen S (2002). Avoiding cross hybridization by choosing nonredundant targets on cDNA arrays. Bioinformatics.

[B32] Li F, Stormo GD (2001). Selection of optimal DNA oligos for gene expression arrays. Bioinformatics.

[B33] Xu D, Li G, Wu L, Zhou J, Xu Y (2002). PRIMEGENS: robust and efficient design of gene-specific probes for microarray analysis. Bioinformatics.

[B34] Tolstrup N, Nielsen PS, Kolberg JG, Frankel AM, Vissing H, Kauppinen S (2003). OligoDesign: Optimal design of LNA (locked nucleic acid) oligonucleotide capture probes for gene expression profiling. Nucleic Acids Res.

[B35] Rouillard JM, Zuker M, Gulari E (2003). OligoArray 2.0: design of oligonucleotide probes for DNA microarrays using a thermodynamic approach. Nucleic Acids Res.

[B36] Chen YA, Chou CC, Lu X, Slate EH, Peck K, Xu W, Voit EO, Almeida JS (2006). A multivariate prediction model for microarray cross-hybridization. BMC Bioinformatics.

[B37] Tong W, Harris S, Cao X, Fang H, Shi L, Sun H, Fuscoe J, Harris A, Hong H, Xie Q (2004). Development of public toxicogenomics software for microarray data management and analysis. Mutat Res.

